# O-Polysaccharides of LPS Modulate *E. coli* Uptake by *Acanthamoeba castellanii*

**DOI:** 10.3390/microorganisms11061377

**Published:** 2023-05-24

**Authors:** Ying Liu, Gerald Koudelka

**Affiliations:** Department of Biological Sciences, University at Buffalo, Buffalo, NY 14260, USA; yliu226@buffalo.edu

**Keywords:** lipopolysaccharide, O-polysaccharide, *A. castellanii*, protozoan predation

## Abstract

Protozoan grazing is a major cause of bacterial mortality and controls bacterial population size and composition in the natural environment. To enhance their survival, bacteria evolved many defense strategies to avoid grazing by protists. Cell wall modification is one of the defense strategies that helps bacteria escape from recognition and/or internalization by its predators. Lipopolysaccharide (LPS) is the major component of Gram-negative bacterial cell wall. LPS is divided into three regions: lipid A, oligosaccharide core and O-specific polysaccharide. O-polysaccharide as the outermost region of *E. coli* LPS provides protection against predation by *Acanthamoeba castellanii*; however, the characteristics of O-polysaccharide contribute to this protection remain unknown. Here, we investigate how length, structure and composition of LPS affect *E. coli* recognition and internalization by *A. castellanii*. We found that length of O-antigen does not play a significant role in regulating bacterial recognition by *A. castellanii*. However, the composition and structure of O-polysaccharide play important roles in providing resistance to *A. castellanii* predation.

## 1. Introduction

The gram-negative bacterial cell wall consists of two lipid bilayers, i.e., the inner membrane and the outer membrane, which are separated by the peptidoglycan layer and the aqueous periplasm [1]. The inner (or cytoplasmic) membrane is composed of glycerophospholipid (PL). Unlike the inner membrane, the outer membrane of gram-negative bacteria is an asymmetrical lipid bilayer. The outer membrane is composed of glycerophospholipid (PL) and lipopolysaccharide (LPS), which are only present on the surface-exposed outer leaflet [2]. LPS consists of three components: lipid A, oligosaccharide core (OS) and O-specific polysaccharide, which is also known as O-antigen. The lipid A moiety consists of a glucosamine disaccharide, which is substituted with fatty acids, that anchors the LPS onto the outer membrane [3]. Lipid A is glycosylated with an oligosaccharide core, which provides the attachment site for a long chain of O-antigenic polysaccharide. The structure and composition of lipid A and inner core sugars of OS are highly conserved. The structure and composition of the outer core of OS displays limited variation. For example, only five core types are found in *E. coli* and two are found in *Salmonella* [4]. In contrast, the O-antigen domain consists of repeating units of one or more sugar residues and is highly diverse, displaying a remarkable range in composition and structure. More than 180 different O-antigens were defined in *E. coli* [5] and 46 were defined in *Salmonella* [6].

Conservation of the structure of lipid-A and inner core OS is believed to be influenced by their crucial role in preserving the integrity of the outer membrane. In particular, the LPS substructure consisting of 3-deoxy-D-manno-oct-2-ulosonic acid (Kdo)2–Lipid A is essential for the growth of the majority of gram-negative bacteria [7,8], with a few exceptions [9,10]. In contrast, the diversity of the external parts of LPS, particularly O-polysaccharide, is likely due to its interaction with other elements in the environments where bacteria reside. This diversity may have a protective function, such as defending against bacterial predation by protozoa, facilitating bacterial escape from the host immune system and/or regulating interactions with other cells and bacteriophages [11,12,13].

Despite the high diversity in the composition and structure of O-antigen, all are synthesized and assembled through one of three pathways: the Wzx/Wzy-dependent pathway [14], the ABC transporter-dependent pathway [15,16] or the synthase-dependent pathway [17]. These three pathways all start with the formation of undecaprenol diphosphate (und-PP)-linked sugar through initial transferase at the cytoplasmic leaflet of inner membrane. Most *E. coli* O-antigens are synthesized via the Wzx/Wzy pathway [5]. In this pathway, following initiation, sugar residues are sequentially added via glycosyltransferases to form the Und-PP-linked O units. The Und-PP-linked O units are then transported across the inner membrane via flippase Wzx [18]. Polymerization of new O-polysaccharides occurs in the periplasmic face of the inner membrane via polymerase Wzy [18,19]. The chain lengths of O-antigens vary but are distributed about a length close to the modal value. Wzz, which is a member of the polysaccharide co-polymerase family, regulates the center of the modal distribution of the number of O units found in the O-antigen polymer.

Predation of bacteria is a major cause of bacterial mortality [20] and, therefore, controls the structure and activity of microbial communities in many ecosystems [21,22,23]. *Acanthamoeba castellanii*, which is a free-living protist, is the main consumer of bacteria. Previous studies in our lab showed that predation of *E. coli* by *A. castellanii* does not require the presence of O-antigen. However, the type of O-antigen present on the bacterial surface modulates bacterial recognition by *A. castellanii* [13]. For example, *E. coli* EDL933 that bears O157 O-antigen can be recognized and consumed, whereas ATCC11775, which is an *E. coli* strain that bears O1, is not recognized or consumed by *A. castellanii* [13]. Consistent with the importance of O-antigen diversity in resisting predation, intestinal protozoa recognize antigenically diverse *Salmonella* with different efficiencies, and their ability of prey discrimination is seemingly solely determined based on the identity of the O-antigen [11].

We wished to explore how the structure and sequence of the O-polysaccharide regulate the efficiency of protist predation. For this study, we first examined whether the length of O-polysaccharide is required for O1 O-antigen to provide the resistance against *A. castellanii* using *E. coli* strain ATCC11775 and ATCC11775Δ*wzzb*. We also examined how O-antigen affect *A. castellanii* predation through assessing the ability of multiple other LPS O-antigens to inhibit uptake by *A. castellanii* of MG1655, which is a K12 strain that lacks O-antigen. We found that O-antigen length does not significantly affect its recognition by *A. castellanii*. However, O-antigen composition and structure play important roles in regulating bacterial uptake by *A. castellanii*.

## 2. Materials and Methods

### 2.1. Strains, Plasmids and Chemicals

*Acanthamoeba castellanii* was a gift from Wendy Trzyna, Marshall University. *E. coli* K12 strain MG1655 was purchased from the Coli Genetic StockCenter. *E. coli* strain ATCC11775 was purchased from the American Type Culture Collection (Manassas, VA, USA). *E. coli* prototype R1(F470), CWG634(O9), CWG1010(O9 deficient), CWG636(O8) and CWG1008(O8 deficient) were gifts from Chris Whitfield, University of Guelph, Canada [24]. LPS were obtained from the following *E. coli* strains: HUSEC4(O157), HUSEC5(O55), HUSEC11(O111), HUSEC12(O73), HUSEC28(128), HUSEC30(O98) and HUSEC39(O76), which are part of the Collection of Hemolytic Uremic Syndrome-Associated Enterohemorrhagic *Escherichia coli* [25]. James Hurst, who works at Washington State University, provided the plasmid-encoding GFP. pKD3 was obtained from Addgene. pKD46RecX was a gift from Michael Berger, University of Münster. pUC18 was used to construct plasmid-carrying *manA* gene from MG1655 (Addgene plasmid # 50004; RRID:Addgene_50004. The *manA* gene fragment was amplified from MG1655 using the following primers: BamHI-manA-Forward: 5′-cgcggatcctatgcaaaaactcattaactcagtgc-3′ and HindIII-manA-Reverse: 5′-cctttaataagcttagcaagagatg-3′. The *manA* gene was inserted between BamHI and HindIII on pUC18.

### 2.2. Purification of LPS

Purification of LPS was performed as described previously [13,26]. In brief, 15 mL overnight culture of bacteria was washed and resuspended in 65 °C water. The LPS molecules were purified via hot phenol extraction. The samples were treated with 0.1 mg/mL DNase I, 0.1 mg/mL RNase A and 0.1 mg/mL Proteinase K during dialysis. Depending on the resolving power needed, LPS purity was assessed via electrophoresis on 12% or 13% SDS-PAGE followed by silver staining. No residual protein or nucleic acids were detected in the final preparation. Figure S1 shows SDS-PAGE purity assessments of all LPS used in this study. The yield of LPS was determined via limulus amebocyte lysate (LAL) assay using the ToxinSensor^TM^ Chromogenic LAL Endotoxin Assay Kit from GenScript USA Inc. (Piscataway, NJ, USA).

### 2.3. Cultivation and Harvesting of A. castellanii

*A. castellanii* cells were grown in 5 mL ATCC Medium 712: Peptone-yeast extract-glucose medium (2% proteose peptone, 0.1% yeast extract, 0.1 M glucose, 4 mM MgSO_4_-7H_2_O, 0.4 mM CaCl_2_, 0.1% sodium citrate dihydrate, 0.05 mM Fe(NH_4_)_2_(SO_4_)_2_-6H_2_O, 2.5 mM Na_2_HPO_4_-7H_2_O, 2.5 mM KH_2_PO_4_) at 30 °C in flat bottom 25 mL tissue culture flasks. After incubation without agitation for 4 days, the growth media were removed from the flasks and replaced with 5 mL of fresh media. The flasks were cooled on ice for 20 min to detach *A. castellanii* from the bottom of the flask, and the cells were collected for either passaging or experimentation. Stocks of *A. castellanii* were prepared and stored at 4 °C according to the method described in [27]. *A. castellanii* cells intended for the predation assay were harvested via centrifugation at 300×g at room temperature, washed via Pages Amoeba Saline (PAS) [27,28] for three times and resuspended in PAS.

### 2.4. Chromosomal Gene Inactivation in ATCC11775

ATCC11775Δ*wzzb* was created using phage λ Red recombinase, as described in [29]. The N-terminal deletion primers were composed of an 80-nt 5′ extension inclusive of the translation initiation codon. The C-terminal deletion primers contained a 21-nt segment representing the C-terminal region, a translation termination codon and a downstream 59-nt sequence. These primers were used to amplify the DNA fragment of chloramphenicol-resistant gene from pKD3: ATCC11775-wzzB-F: 5′-atcaattatcctatagcattcatgaggattatcgctaaactatgcggacttggaaatttccgtcagttagggtaatgatggtgtaggctggagctgcttc-3′ and ATCC11775-wzzB-R: 5′-tccggcaaaaaaacgggcaaggtgcaccaccctgccctttttctttaaaaccgaaaagattacttcgcgttgtaattgcgatgggaattagccatggtcc-3′. ATCC11775 carrying the Red helper plasmid pKD46RecX was grown in 30 mL with 100 μg/mL ampicillin at 30 °C until an OD_600_ of 0.1. Following the addition of 100 mM L-arabinose, ATCC11775 was grown at 30 °C to an OD_600_ of 0.6. Cells were washed using ice-cold 10% glycerol for three times and resuspended in sterile ddH_2_0. A total of 50 μL of cells was mixed with 600 ng of the PCR fragment in an ice-cold 0.2 cm cuvette. Cells were electroporated at 2.5 kV with 25 mF and 200 Ω, which was immediately followed by the addition of 1 mL of SOC medium (2% Bacto Tryptone, 0.5% yeast extract, 10 mM NaCl, 2.5 mM KCl, 10 mM MgCl_2_, 10 mM MgSO_4_, 20 mM glucose). After overnight incubation at 37 °C, it was spread onto agar plate with 15 μg/mL chloramphenicol to select CmR transformants at 37 °C. Verification of correct chromosomal insertion was carried out via PCR with chloramphenicol gene specific primer and locus specific primer. Primers used for verification were ATCC11775-wzzB-VF: 5′-atcgctaaactatgcggact-3′ and ATCC11775-wzzB-VR: 5′-gatatgggatagtgttcacccttgtta-3′.

### 2.5. Predation Assay

Predation assay was performed as described previously [30]. In brief, proteose peptone glucose (PPG) agar plates (1% proteose peptone, 1% glucose) were separately seeded with 100 μL of saturated cultures (10^9^ cells/mL) of different *E. coli* strains and incubated for 1 h at 37 °C. Subsequently, 10^5^ *A. castellanii* trophozoites in 10 μL were spotted on each plate. Each plate was sealed with parafilm and incubated at 28.5 °C for 5 days for ATCC11775 and its mutant or 4 days for CWG634 and CWG1010. After incubation, the plates were photographed and the size of plaque formed by *A. castellanii* consumption on each plate was calculated using ImageJ [31]. Each measurement was performed in quadruplicate and averaged. The data shown represent at least 3 independent quadruplicate measurements.

### 2.6. Competition Assay with Purified LPS

In total, 400 µL of freshly harvested 4–6 day old *A. castellanii* were aliquoted into each well of a 24-well polystyrene tissue culture plate and allowed to incubate overnight at 30 °C. After removing the planktonic cells suspended in medium, the *A. castellanii* cells adhered to the bottom of each well were washed three times at room temperature with Pages Amoeba Saline (PAS) [27,28] and suspended overnight in 300 μL PAS. *E. coli* MG1655 expressing GFP were grown in LB supplemented with 30 ug/mL kanamycin. Bacteria were washed three times at room temperature with PAS. *A. castellanii* in PAS were incubated with 4 × 10^6^ EU/mL purified LPS at room temperature for 1 h and co-cultured with 6 × 10^8^ *E. coli* MG1655 expressing GFP for 1 h at room temperature, followed by 2 h incubation at 30 °C. After incubation, the *A. castellanii* were washed with PAS three times, and the plates were placed on ice for 20 min to release *A. castellanii* cells for measurement. The fraction of GFP-positive *A. castellanii* was measured using the LUNA-FL™ Dual Fluorescence Cell Counter (Logos Biosystems, Annandale, VA, USA).

### 2.7. Statistical Methods

Error bars in the figures were standard deviations of at least three biological replicate experiments, with each containing at least two technical replicates. Data were analyzed via unpaired two-tailed *t*-tests with Welch’s correction and/or the one-way analysis of variance (ANOVA), followed by post hoc implementation of Tukey’s multiple comparisons correction to determine the statistical significance of pairwise or multiplex comparisons.

## 3. Results

### 3.1. O1 Antigen Blocks Bacterial Consumption by A. castellanii

Previous research suggested that particular O-antigens negatively regulate the efficiency of the process of bacterial recognition and consumption by *A. castellanii*, i.e., ATCC11775, an *E. coli* strain that bears O1, is not recognized or consumed by *A. castellanii* [13]. To further investigate how O1 O-antigen blocks the recognition and uptake of ATCC11775 by *A. castellanii*, we hypothesized that the length of O1 O-antigen may inhibit recognition of *E. coli* by *A. castellanii* through generating the physical distance between the bacterial determinant Kdo and the *A. castellanii* surface receptor: mannose-binding protein (aMBP).

**Figure 1:**
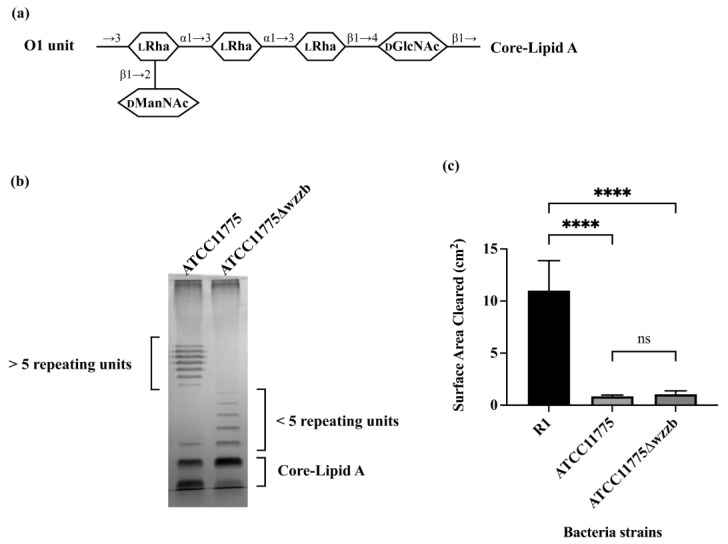
Predation resistance of O1 antigen is independent of its chain length. (**a**) Serotype of ATCC11775 is O1. Shown in panel A is the structure of one repeating unit of O1a [5,35]. Identities of component sugars are indicated inside hexagons (_D_GlcNAc: N-acetyl-D-glucosamine; _L_Rha: L-rhamnose; _D_ManNAc: N-acetyl-D-mannosamine). O1a has a tetra-saccharide backbone that contains one D-GlcNAc as initial sugar proximal to core-lipid A and three L-Rha residues. One D-ManNAc side-branch is linked at outermost L-Rha. (**b**) Visualization of LPS on 12% SDS-PAGE, followed by silver staining, is shown in panel B. LPS was extracted using hot phenol from *E. coli* strain ATCC11775 and its mutant strain ATCC11775Δwzzb. Note bands at very top of gel are from nonspecific staining of interface between stacking and separating gels. (**c**) Panel C shows effect of O-polysaccharide length on *A. castellanii* predation of *E. coli. A. castellanii* (10^6^ cells in a volume of 10 μL) were separately spotted on PPG plates seeded with 10^8^ cells of *E. coli* strains R1, ATCC11775 and ATCC11775Δwzzb. *A. castellanii* were allowed to grow on these plates for 5 days at 28.5 °C. If *A. castellanii* graze bacteria on plate, it will form a plaque on bacterial lawn. Sizes of plaques were determined using ImageJ. Error bars represent standard deviations from at least three independent quadruplicate measurements. Statistical analysis was performed using a one-way analysis of variance (ANOVA), with a subsequent application of Tukey’s multiple comparisons correction. Pairwise comparisons were conducted using adjusted *p* value to determine statistical significance. **** denotes *p* < 0.0001, while ns denotes a lack of statistical significance (*p* > 0.05).

To test this idea, we manipulated the length of the O1 antigen chain. O1 is synthesized through the Wzx/Wzy-dependent pathway. In this pathway, Wzz functions as the chain length regulator and helps define the O-antigen chain length distribution [32]. Manipulation of Wzz can modulate the distribution of O-antigen chain length [33]. We, therefore, constructed an in-frame disruption of Wzz in ATCC11775 through inserting a chloramphenicol resistant cassette using phage λ Red recombinase [34]. The distribution of LPS chain length of ATCC11775 and ATCC11775Δ*wzzb* was evaluated via silver-stained SDS-PAGE (Figure 1b). LPS purified from ATCC11775 displays a distribution of bands located in the upper region of the gel, which is indicative of a high molecular weight O-antigen. Meanwhile, ATCC11775Δ*wzzb* only displays distribution of bands in the lower region of the gel, just above the bands representing core-lipid A. This observation shows that, as expected, the Δ*wzzb* mutation blocked the formation of high molecular weight O1 O-antigen. Thus, without Wzz protein, the chain length regulator ATCC11775Δ*wzzb* is no longer capable of synthesizing the full length of O1 O-antigen and maintaining its preferred modal distribution. ATCC11775Δ*wzzb* still possesses functional Wzz flippase and Wzy polymerase,; therefore, a few repeating O-units are added onto its LPS, giving rise to the short length O-antigen chain visible on SDS-PAGE (Figure 1b). These findings are also consistent with previous research into the role of WzzB in modulating O-antigen chain length [33].

To determine how O1 O-antigen chain length affects bacterial uptake by *A. castellanii*, a solid phase assay was utilized to examine the ability of *A. castellanii* to consume bacteria growing on proteose peptone glucose (PPG) agar plates. In this assay, if bacteria seeded on a PPG agar plate are consumed by *A. castellanii*, a plaque forms in the bacterial lawns. The extent of bacterial consumption by *A. castellanii* is directly correlated with the size of the resulting plaque. For this experiment, ATCC11775 and ATCC11775Δ*wzz*b mutant strain were separately seeded onto PPG agar plates. Subsequently, equal numbers of *A. castellanii* were spotted at the center of each plate. The *A. castellanii* were allowed to grow on these plates for 5 days at 30 °C prior to measurement of plaque size. Since ATCC11775 contains a R1 OS region, we also examined the ability of *A. castellanii* to consume the O-antigen deficient *E. coli* R1 prototype strain as control to monitor the efficiency of bacterial consumption by *A. castellanii* when no O-antigen is present.

The size of the *A. castellanii* plaque formed on ATCC11775 was significantly smaller than that formed on the O-antigen-deficient, R1 prototype strain (Figure 1c). In fact, the size of the *A. castellanii* plaque formed on lawn of ATCC11775 remained unchanged over the course of the entire experiment, indicating that *A. castellanii* did not consume this strain. This result shows that presence of the O1 O-antigen “blinds” *A. castellanii* to the presence of ATCC11775, a finding that is consistent with our previously reported results [13]. Importantly, we found that the size of the plaques formed on ATCC11775 and ATCC11775Δ*wzzb* were identical. Thus, neither ATCC11775, which displays full-length O1, nor ATCC11775Δ*wzzb*, which contains very few O1 units, were consumed by *A. castellanii*. This result clearly indicates that the inability of *A. castellanii* to recognize ATCC11775 as prey is independent of the number of repeating units in the O1 O-antigen, instead being due to the composition and/or structure of the O1 unit.

### 3.2. O8 and O9 Antigens Enhance Bacterial Consumption by A. castellanii

**Figure 2:**
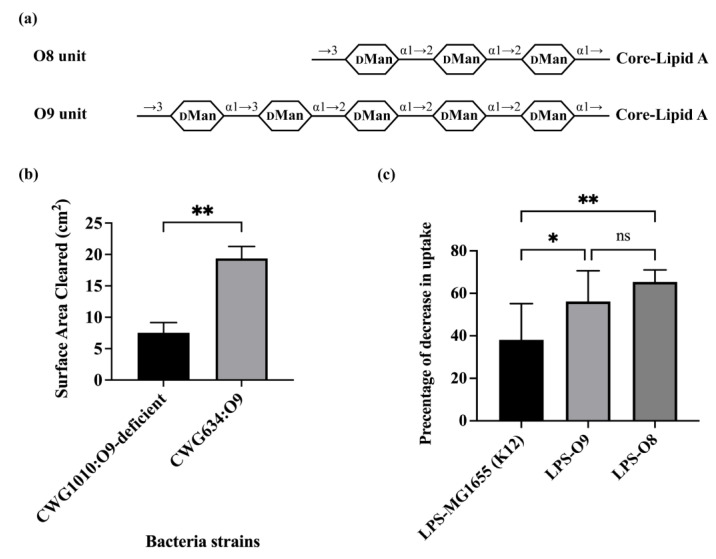
Effect of mannose containing O-antigen on bacterial uptake by *A. castellanii*. (**a**) O-unit structures of O9 and O8. Shown in diagram of panel A are structures of repeating unit of O9a and O8 [5,37,38]. Identities of component sugars are indicated inside hexagons (_D_Man: D-Mannose). O9a has a linear backbone consisting of five D-mannose residues. O8 has a trisaccharide backbone that is composed of three D-mannose residues. No side-branch found in structure of either. As O9a and O8 were synthesized via ABC transporter pathway, GlcNAc only appeared once as primer proximal to core-lipid A (not shown in this diagram) in their LPS. (**b**) *A. castellanii* predation on *E. coli* O9 and O9-deficient strains. *A. castellanii* (10^5^ cells in a volume of 10 μL) were separately spotted on PPG plates seeded with 10^8^ cells of *E. coli* strains CWG634 and CWG1010, with both carrying p:manA. *A. castellanii* were allowed to grow on these plates for 4 days at 28.5 °C. Error bars represent standard deviations from at least three independent quadruplicate measurements. Unpaired two-tailed *t*-tests with Welch’s correction were performed to determine statistical significance. (**c**) Effect of purified LPS O9 and O8 on *E. coli* recognition and internalization by *A. castellanii*. Purified LPS from *E. coli* MG1655, CWG634 (O9a) and CWG636 (O8) were separately incubated with *A. castellanii* at room temperature for 1 h. *A. castellanii* cells were then co-cultured with MG1655 expressing GFP and processed for measurement, as shown in Section 2. Fraction of GFP-positive *A. castellanii* was measured via LUNA-FL™ Dual Fluorescence Cell Counter. Shown in this graph, it is decrease in internalization as a percentage of total uptake. Error bars represent standard deviations from at least five independent experiments, with each having at least two technical replicates. One-way analysis of variance (ANOVA), followed by post hoc implementation of Tukey’s multiple comparisons correction. Comparisons to determine statistical significance were made using the adjusted *p*-value. **: *p* < 0.01; *: *p* < 0.05; ns: not significant *p* > 0.05.

The results in Figure 1 show that O-antigen can block bacterial consumption by *A. castellanii*. We wished to determine whether other O-antigens could have the converse effect, enhancing bacterial consumption by *A. castellanii*. We showed previously that recognition and consumption of *E. coli* by *A. castellanii* is mediated by the mannose-binding protein (MBP) present on the surface of *A. castellanii* [13]. This observation suggests that the presence of mannose in the O-antigen could enhance bacterial consumption by *A. castellanii*. Of the more than 180 known *E. coli* O-antigens, only O8 and O9 O-antigens comprise a linear repeating unit composed of one single monosaccharide, i.e., mannose (Figure 2a). These two O-antigens differ only in the number of mannoses and the linkages between these mannoses present in each repeated unit.

To examine the impact of a mannose containing O-antigen on the efficiency of bacterial consumption by *A. castellanii*, we compared the ability of *A. castellanii* to consume the O9 O-antigen expressing strain CWG634 with its O-antigen deficient strain, i.e., CWG1010 [24]. CWG634 does not produce O9 O-antigen in the absence of added mannose because it is deficient in *man*A (mannose-6-phosphate isomerase). However, because mannose competes with bacterial recognition of *E. coli* by *A. castellanii*, to complete this experiment, we used strains that were transformed with a plasmid that expresses *man*A, which allows CWG634 to produce O9 in the absence of added mannose. We found that *A. castellanii*formed plaque on CWG634-pmanA was nearly twice the size of the plaque formed on CWG1010. This result indicates that *A. castellanii* consumed CWG634 with O9 O-antigen at a significantly higher rate of than its O-antigen deficient counterpart CWG1010 (Figure 2b).

We wished to confirm the observation that a mannose-containing O-antigen enhances bacterial consumption by *A. castellanii* through repeating this experiment with the O8-containing strain CWG636 [24]. Unfortunately, CWG636 is incapable of growing on the plates used for the solid phase predation assay. Therefore, we compared how well O8- and O9-containing LPS purified from CWG636 and CWG634, respectively (Figure S1), competed with the uptake of GFP-labeled MG1655, which is an O-antigen deficient K12 strain, by *A. castellanii*. We also compared the effect of LPS purified from MG1655 on bacterial consumption to examine the impact of LPS-lacking O-antigen.

In this experiment, we first separately incubated *A. castellanii* without or with equal amounts of the three different purified LPS molecules (see Methods and Materials for details on purification and quantification of LPS) for one hour at room temperature. These *A. castellanii* were subsequently co-cultured with MG1655 expressing GFP for one hour at room temperature, followed by two hours at 30 °C. Bacterial consumption by *A. castellanii* was determined by measuring the fraction of *A. castellanii* containing GFP-labeled bacteria. If the purified LPS was recognized by *A. castellanii*, it could compete with the GFP-labeled MG1655 and, therefore, decrease the bacterial uptake by *A. castellanii*. As compared to the absence of any added LPS, LPS purified from MG1655 decreased the fraction of *A. castellanii* containing GFP-labeled bacterial by 38.10% (Figure 2c). However, purified LPS from CWG634 (O9) and CWG636 (O8) inhibited the bacterial uptake by 56.14% and 65.38%, respectively, which was significantly higher than the figure for purified LPS from MG1655 (Figure 2b). LPS from O8 and O9 contain the R1 oligosaccharide (OS) region. The difference between purified LPS from CWG634 (O9) and CWG636 (O8) is not significant. Our previous observations show that LPS lacking O-antigen but containing R1 OS are more poorly recognized by *A. castellanii* than are those bearing K12 OS [36] This observation explains why LPS-K12 inhibits more effectively than LPS-O8 and O9. Regardless, taken together, the results in Figure 2 indicate that the O8 and O9 antigens interact identically with *A. castellanii*, while *E. coli* that express O-antigens that are composed solely of mannose are consumed much more efficiently than bacteria that do not express this type of O-antigen.

### 3.3. Role of O-Antigen Structure in Regulating Efficiency of Bacterial Predation by A. castellanii

The results in Figure 1 and Figure 2 clearly show that the presence and type of O-antigen present on the surface of *E. coli* can remarkably affect the bacterial predation efficiency of *A. castellanii*. It is unclear whether O-antigen structure, its composition or a combination of both factors play a role in regulating this process. We wished to further define the structure and compositional determinants on O-antigen that impact the ability of *A. castellanii* to recognize and consume its bacterial prey. Therefore, we obtained LPS from multiple *E. coli* strains in the HUSEC collection [25]. We used LPS from *E. coli* in this collection, since it provided ready access to a variety of strains possessing different O-antigens attached to the same R3 OS type.

The HUSEC strains all contain a prophage that encodes Shiga toxin. Shiga toxin encoding *E. coli* strains kill *A. castellanii*, meaning that their consumption cannot be measured directly in solid phase assay. Instead, to determine how well these LPS were recognized by the amoeba, as for the experiment shown in Figure 2c, we examined how effectively purified LPS from each of these strains competed with the uptake of GFP-labeled *E. coli* by *A. castellanii*. Unfortunately, the GFP-expressing prototype R3 strain lacking O-antigen grows poorly on PPG plates; hence, we were unable to use this strain as prey in this assay. However, we showed previously [36] that *A. castellanii* recognizes strains lacking O-antigen but containing either R3 or K12 OS regions equally well. *E. coli* K12 strain MG1655 grows well on PPG plates. The similarity in *A. castellanii* uptake in the R3 prototype and K12 strains allows us to attribute any observed differences in the ability of the various LPS types to compete with uptake of *E. coli* by *A. castellanii* to differences in the structure and/or composition of the added LPS.

**Figure 3:**
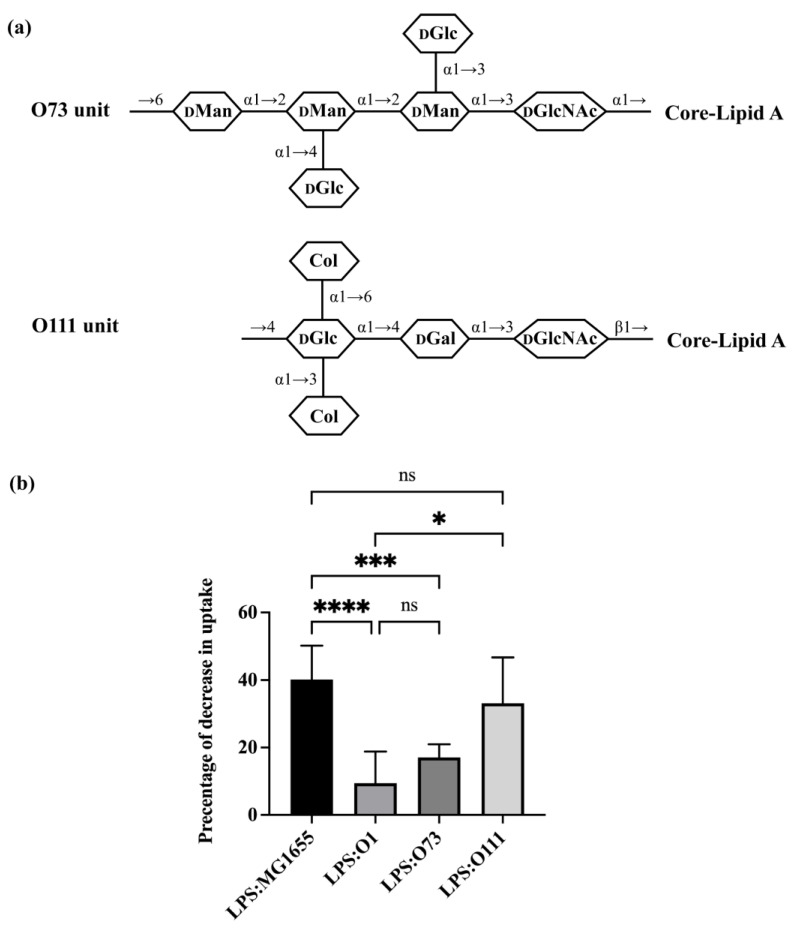
Effect of O-antigen with two side-branches on *E. coli* consumption by *A. castellanii*. (**a**) Structures of one repeating unit of O73 and O111 [5,39,40]. Identities of component sugars are indicated inside hexagons (Col: Colitose; _D_Gal: D-Galactose; _D_Glc: D-Glucose; _D_GlcNAc: N-acetyl-D-glucosamine; _D_Man: D-Mannose). Both have GlcNAc as initial sugar (proximal to core-lipid A). (**b**) Effect of purified LPS O73 and O111 on *E. coli* recognition and internalization by *A. castellanii*. Purified LPS from *E. coli* MG1655, ATCC11775 (O1), HUSEC24 (O73) and HUSEC11 (O111) were separately incubated with *A. castellanii* at room temperature for 1 h. *A. castellanii* cells were then co-cultured with MG1655 expressing GFP and processed for measurement, as shown in the Section 2 and Figure 2. Error bars represent standard deviations from at least three independent experiments, with each having at least two technical replicates. A one-way analysis of variance (ANOVA) was performed, followed by Tukey’s multiple comparison correction for post hoc analysis. Adjusted *p*-value was used to determine statistical significance of pairwise comparisons. A significance level of *p* < 0.0001 was denoted by ****; ***: *p* < 0.001; *: *p* < 0.05 while comparisons with *p* > 0.05 were considered insignificant and denoted by ns.

The O-unit of O1 is comprised of a tetra-saccharide backbone that is substituted with one side branch (Figure 1a). The O-units of O8 and O9 are unbranched (Figure 2a). To begin to examine the role of branching in regulating bacterial uptake by *A. castellanii*, we examined the effect of adding LPS that have two branches in their O-units (O73 and O111). Less than 20% of all *E. coli* O-antigens have a structure that contains two branches in their O-units [5]. O73 has a tetra-saccharide backbone (GlcNAc-Man-Man-Man) and two side-branch glucose residues (Figure 3a) [39]. The first sugar in the backbone is GlcNAc, which is the most common first sugar in any given *E. coli* O-antigen [5]. The rest of the backbone consists of mannose, which is the monosaccharide favored by MBP (Figure 2). The two side-branch glucose moieties are linked to the middle two mannose residues. O111 has a trisaccharide backbone (GlcNAc-Gal-Glc) with two side-branch colitose residues linked to the same glucose residue in the backbone (Figure 3a) [40]. O111 also has GlcNAc as its first sugar in the backbone.

Adding purified LPS (Figure S1) bearing the O73 O-antigen had only a slight (~17%) inhibitory effect on bacterial uptake by *A. castellanii* (Figure 3b). The effect of O73 on bacterial uptake was significantly lower than LPS purified from MG1655 (40.2%), but similar to that of the O1 LPS purified from ATCC11775 (9.4%). This finding indicates that *A. castellanii* only weakly recognizes O73, and that this O-antigen inhibits recognition of the bacterial determinant in the OS region. Moreover, the structure of O73 apparently overcomes any *A. castellanii* binding advantage conferred by the mannose residues present in O73. In contrast, purified O111 LPS effectively competes with the GFP-labeled MG1655, decreasing its uptake by >33%. Together, these results indicate that the presence of a two side-branch structure alone is not the sole determinant that blocks *A. castellanii* recognition of LPS. Apparently, the precise arrangement and/or the composition of the side branch plays an important role in regulating this process.

**Figure 4:**
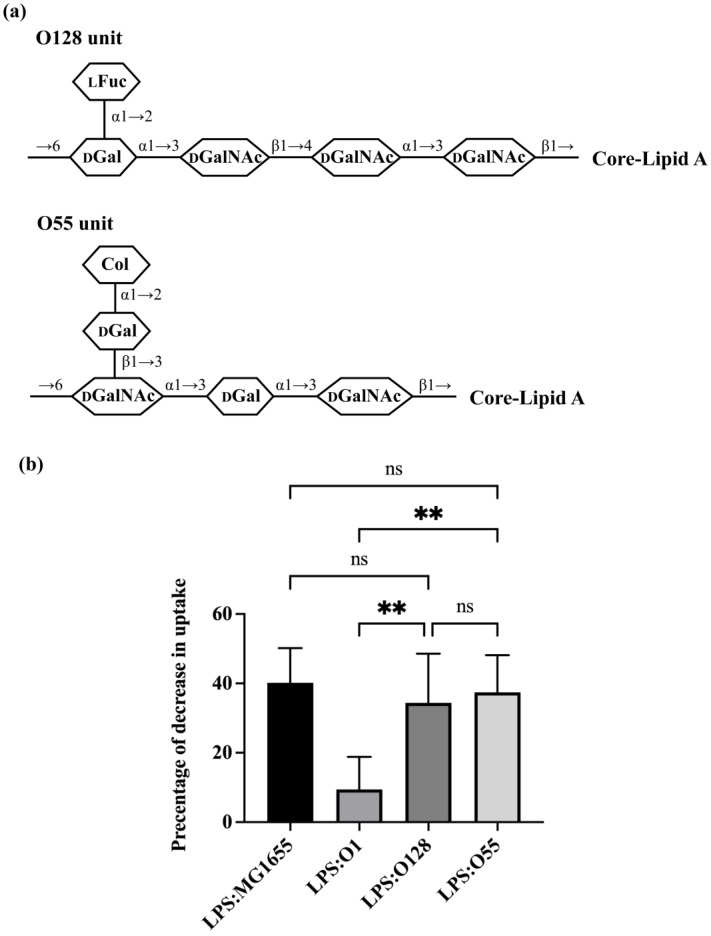
Effect of O-antigen with one side branch on *E. coli* recognition by A. castellanii. (**a**) Structures of one repeating O-unit of O128 and O55 [5,41,42]. Identities of component sugars are indicated inside hexagons (Col: Colitose; _L_Fuc: L-Fucose; _D_Gal: D-Galactose; _D_GalNAc: N-acetyl-D-galactosamine; _D_GlcNAc: N-acetyl-D-glucosamine). Both of them have GalNAc as initial sugar (proximal to core-lipid A). (**b**) Effect of purified LPS O1128 and O55 on *E. coli* recognition and internalization by *A. castellanii*. Purified LPS from *E. coli* MG1655, ATCC11775 (O1), HUSEC28 (O128) and HUSEC5 (O55) were separately incubated with *A. castellanii* at room temperature for 1 h. *A. castellanii* cells were then co-cultured with MG1655 expressing GFP and processed for measurement, as shown in the Section 2. The fraction of GFP-positive *A. castellanii* was determined via LUNA-FL™ Dual Fluorescence Cell Counter. As shown in this graph, it is decrease in internalization as a percentage of total uptake. Error bars represent standard deviations from at least five independent experiments, each of which has at least two technical replicates. One-way analysis of variance (ANOVA), followed by post hoc implementation of Tukey’s multiple comparisons correction. Comparisons to determine statistical significance were made using adjusted *p*-value. **: *p* < 0.01; ns: not significant *p* > 0.05.

To further explore this issue, we examined the efficiency with which LPS-bearing O-antigens containing one side-branch blocked *E. coli* uptake by *A. castellanii*. For this experiment, we examined LPS containing O128 and O55 (Figure S1). O128 (Figure 4a) contains a tetra-saccharide backbone with one side-branch, which is the most common O-antigen structure in *E. coli* [5], while O55 (Figure 4a) has a trisaccharide backbone with one disaccharide side-branch. This latter arrangement combines two of the most uncommon features among *E. coli* O-antigens. Both O128 and O55 efficiently competed with the GFP-labeled MG1655, decreasing its uptake by 34.39% and 37.42%, respectively (Figure 4b). The effect of these LPS variants on bacterial uptake are similar to that of LPS from MG1655. These findings indicate that both O128 and O55 are recognized by *A. castellanii* and would not confer resistance against *A. castellanii* predation. Since O1, which also contains one side-branch, does confer such resistance and does not block bacterial uptake, these findings also show that solely using the one side-branch structure itself is insufficient to block LPS recognition by *A. castellanii*, as well as that either (1) where the side-branch is located in the structure of O-antigen, or (2) the composition of O-antigen, either in the backbone or the side-branch, impacts LPS recognition by *A. castellanii*.

**Figure 5:**
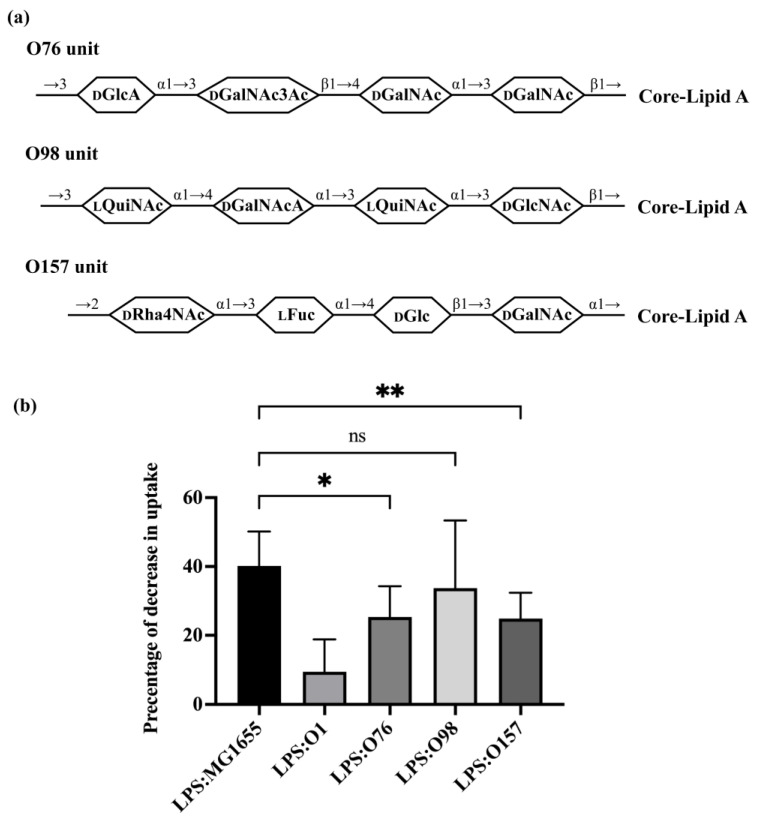
Effect of O-antigen with one side branch on *E. coli* recognition by *A. castellanii*. (**a**) Structures of one repeating unit of O76, O98 and O157 [5,43,44,45]. Identities of component sugars are indicated inside hexagons (Col: Colitose; _L_Fuc: L-Fucose; _D_Gal: Galactose; _D_GalNAc: N-acetyl-D-galactosamine; _D_GalNAcA: 2-acetamido-2-deoxy-D-galacturonic acid; _D_GlcA: D-Glucopyranuronic acid; _D_GlcNAc: N-acetyl-D-glucosamine; _L_QuiNAc: 2-acetamido-2-deoxy-L-quinovose; _D_Rha4NAc: 4-acetamido-4-deoxy-α-D-rhamnopyranosyl). All of them have a linear tetra-saccharide backbone without any side-branch. Both O76 and O157 have GalNAc as an initial sugar (proximal to core-lipid A), while O98 has GlcNAc as an initial sugar. (**b**) Effect of purified LPS O76, O98 and O157 on *E. coli* recognition and internalization by *A. castellanii*. Purified LPS from *E. coli* MG1655, ATCC11775 (O1), HUSEC39 (O76), HUSEC30 (O98) and HUSEC4 (O157) were separately incubated with *A. castellanii* at room temperature for 1 h. *A. castellanii* cells were then co-cultured with MG1655 expressing GFP and processed for measurement, as shown in Section 2. Fraction of GFP-positive *A. castellanii* was determined via LUNA-FL™ Dual Fluorescence Cell Counter. As shown in this graph, it represents decrease in internalization as a percentage of total uptake. Error bars represent standard deviations from ≥4 independent experiment, with each having at least two technical replicates. The one-way analysis of variance (ANOVA), followed by post hoc implementation of Dunnett’s multiple comparisons correction. Comparisons to determine statistical significance were made using the adjusted *p* value. **: *p* < 0.01; *: *p* < 0.05; ns: not significant *p* > 0.05.

Having explored the role of branching in regulating recognition of O-antigen LPS by *A. castellanii*, we more directly investigated the role of O-antigen composition in this process. For this experiment, we investigated the ability of three different O-antigens, i.e., O76, O98, O157, each of which has a linear structure but differing carbohydrate composition (Figure 5a), to modulate uptake of bacteria. Purified LPS from both O76 and O157 (Figure S1) compete with the GFP-labeled MG1655, decreasing uptake of MG1655 by 25.30% and 24.88%, respectively (Figure 5b). These LPS molecules were more effective competitors than the O1 LPS purified from ATCC11775, but significantly weaker competitors than LPS from MG1655. Adding purified LPS containing the O98 antigen decreased uptake of MG1655 by 33.73%, which is an amount similar to that affected due to LPS from MG1655. Together, these results emphasize the important role that the sugar composition of O-antigen backbone plays in regulating bacterial recognition and uptake by *A. castellanii*.

## 4. Discussion

O-antigen is an important bacterial virulence factor. Loss of O-antigen makes many pathogens sensitive to serum or otherwise seriously impairs their virulence [46,47,48,49,50,51,52]. In many cases, the length of O-polysaccharide influences their virulence [47]. However, we show here that the resistance of bacteria bearing the O1 O-antigen to *A. castellanii* predation was unaffected by the length of O-antigen polysaccharide chain (Figure 1c). This finding indicates the predation resistance provided by O1 relies on the composition and/or structure (side-branch), rather than the physical distance of *A. castellanii* from the bacterial cell surface. This conclusion is consistent with previous research showing that certain sugar residues in the outer core regulate the bacterial uptake by *A. castellanii* [36,50], indicating that the composition or structure of even a short chain of oligosaccharide is sufficient to provide resistance against *A. castellanii* predation. However, whether the composition of the O-unit backbone or side-branch of O1 provided such protection remains unknown.

Our finding that O8 and O9 (Figure 2b,c) increased the efficiency of *E. coli* uptake by *A. castellanii* highlights the important role of composition in regulating *E. coli* recognition. Thus, the presence of mannose, a sugar that binds preferentially to the cell surface receptor, i.e., the mannose-binding protein, enhances bacterial consumption by this predator. We suggest that the mannose in these O-antigens provides another binding site for the *A. castellanii* mannose-binding protein, which leads to the observed elevated recognition and consumption of bacteria (Figure 2b,c). However, the simple presence of mannose in the O-antigen does not always confer preferential uptake by *A. castellanii*. For example, O73, which contains three mannose residues within a doubly branched O-antigen, is only weakly recognized by *A. castellanii*. This finding emphasizes the importance of O-antigen structure.

Previous research indicates the Kdo2 moiety of inner core is the bacterial determinant that allows *A. castellanii* to recognize *E. coli* as prey [36]. Since predation resistance is apparently independent of the length of the O-antigen chain (Figure 1c), the structure and/or composition of O-unit somehow prohibits *A. castellanii* from accessing the Kdo2 determinant and prevents its adherence to, or internalization of, certain *E. coli* strains. Unfortunately, our results do not allow us to completely dissect the role of O-antigen structure and composition in regulating the predation resistance of *E. coli.* However, through comparing the results from our groups of two-branched LPS, one-branched LPS and non-branched LPS, we can gain some insight into how LPS structure and/or composition affects the ability of *A. castellanii* to recognize and internalize bacteria.

Among the O-antigens we examined, O73 and O111 have the most complex structure, with each containing two and one residue side-branches. In O73, the two side-branch sugars are linked to different backbone sugars; however, in O111, the side-branched residues are linked to the same sugar. Purified LPS containing O73 was unable to block uptake of GFP-labeled *E. coli*, indicating that bacterial strains bearing O73 cannot be efficiently recognized by *A. castellanii* and, therefore, would be resistant to *A. castellanii* predation. Among the three O-antigens that contain one side-branch, only O1 is unable to block uptake of GFP-labeled *E. coli* and is, thus, resistant to predation by *A. castellanii*. Since certain one- and two-branched structures are recognized by *A. castellanii*, while others are not, these results indicate that branch structure, per se, is not the dominant determinant of predation resistance. Instead, together, the results in Figure 3 and Figure 4 suggest a more predominant role for O-antigen composition in regulating recognition of bacteria by *A. castellanii*.

Consistent with this conclusion, we find that the ability of *A. castellanii* to recognize each of the three non-branched O-antigens varies with their composition (Figure 5b). Of the three O-antigens examined here, O76 and O157 partially block uptake of GFP-labeled *E. coli*, and are, thus, anticipated to provide some level of protection from *A. castellanii* predation (Figure 5b), whereas O98 efficiently blocks uptake of GFP-labeled *E. coli* and, thus, is anticipated to be highly susceptible to *A. castellanii* predation. O76 consists of two GalNAc residues: one GalNAc3Ac residue, the degree of O-acetylation of which is roughly 70%, and one GlcA residue (Figure 5a) [43]. These modifications mean that O76 is N-acetylated, O-acetylated and negatively charged. In contrast, all sugars in the O98 backbone are N-acetylated and one of them is negatively charged (GalNAcA), and this O-antigen shows a strong ability to block uptake of GFP-labeled *E. coli*, meaning bacteria bearing O98 would be highly susceptible to *A. castellanii* predation (Figure 5b). Together, these findings suggest that the O-acetylation may regulate recognition of *E. coli* by *A. castellanii*. O157 consists of one α-D-Rha4NAc residue, one α-L-Fuc residue, one β-D-Glc residue and one α-D-GalNAc residue [45]. α-D-Rha4NAc is very rare and present only in O157. Rha and L-Fuc are both deoxy sugars that are hexoses without a hydroxyl group at the 6-position. Rha residues were also found in the backbone of O1, although they are in the L configuration. Whether these deoxy sugars play a role in regulating bacterial uptake by *A. castellanii* requires further investigation.

LPS containing either O73 or O1, both of which contain at least on side branch, are not recognized by *A. castellanii*. This finding suggests that structure may play some role in regulating *A. castellanii* recognition of its bacterial prey. Unfortunately, our data do not allow us to discern the relative contributions of structure and composition in *A. castellanii* recognition of O1. However, two other observations support the idea that structure plays some role in regulation recognition of O-antigen by *A. castellanii*. Firstly, the results in Figure 2 show that *A. castellanii* efficiently recognize and predate bacteria containing O-antigens that are comprised only of mannose. Secondly, although the backbone of O73 consists of three mannose residues and one GlcNAc residue, *A. castellanii* do not recognize this O-antigen. The presence of the GlcNAc residue at the first position of the O73 repeat structure is not the cause of this failure to be recognized by *A. castellanii*, since we find no correlation with *A. castellanii* recognition of O-antigen containing either GlcNAc or GalNAc as the initial sugar of the repeating unit. Therefore, the presence of two branching Glc residues on O73 “blind” *A. castellanii* to the presence of mannose in the O-antigen. Thus, the side branches seemingly play an important role in regulating bacterial recognition by *A. castellanii*.

During analysis of our results in the context of O-antigen structure and composition, we noticed that the backbone compositions of O17, O44, O77 and O106 are identical to that of O73. Consistent with this observation, the gene sequences encoding the enzyme responsible for synthesis of these O-antigens in these strains are nearly identical [39,53,54]. However, these O-antigens are decorated with branched substituents whose compositions and identities vary widely. Among the O-antigens in the same group as *E. coli* O73, *E. coli* O77 has a linear tetra-saccharide backbone with no side branch. The *E. coli* O17 and O44 O-antigens are modified through a single side-branch glucose residue but attached at different positions along the backbone. O106 contains two glucose residues as side-branches, which is similar to O73, but the locations where the sugars are linked in the backbone vary. Further investigation of these O-antigens may provide confirmation of the role of glucose side-branches in predation resistance and offer more insight on how the numbers and positions of glucose side-branches play a role in preventing recognition and internalization by *A. castellanii*.

The glycosyltransferases responsible for adding the side branches to the O73 backbone have not yet been identified. However, in the cases where the genes encoding the proteins responsible for adding the side branch sugars were identified (e.g., O44), these genes are atypically not located in, or adjacent to, their O-antigen gene cluster. Instead, they are located elsewhere in the genome. In the case of O44, the enzymes GtrB, GtrA, and WbbG were identified to be responsible for the glucose side-branch in O44 [39]. GtrB is the bactoprenol glucosyltransferase that catalyzes the transfer of glucose from UDP-glucose to bactoprenol phosphate (UndP) to generate UndP-glucose. GtrA is the flippase that translocates UndP-glucose to the periplasmic leaflet of inner membrane. WbbG is the glycosyltransferase that forms the linkage between glucosyl group and mannose in the backbone [39]. The genes encoding these enzymes are associated with a putative prophage, adjacent to attL, located between ybhB and ybhC, which is a normal site for insertion of λ phage [55]. The same pattern of phage-mediated glucosylation was previously discovered and elucidated in *S. flexneri* [56]. *S. flexneri* O antigens of F1–5 have the same O-unit backbone structure and are different because of phage-encoded modifications (glucosylation or/and O-acetylation) [57]. Three type-specific genes, i.e., *gtr*A, *gtr*B, and *gtr*C, are found responsible for the glucosylation in *S. flexneri* and are arranged in one operon [56]. Among these three genes, *gtr*A and *gtr*B are highly conserved in *S. flexneri* and *E. coli* [39]. The locations of these genes led to the hypothesis that the genes encoding these branch-addition glucosyltransfereases were introduced into these strains via temperate bacteriophage [39]. It is well known that for many bacteriophages, the long O-antigen chains found on LPS serve as the essential receptor recognized by their tail spike proteins. Our work here suggests that this phage-mediated bacterial cell wall modification may also give the bacteria evolutionary advantage under the pressure of predation from protists and phages.

## Data Availability

The data presented in this study are available in this article.

## Supplementary Materials

The following supporting information can be downloaded at: https://www.mdpi.com/article/10.3390/microorganisms11061377/s1, Figure S1: Silver-stained gel displaying purified LPS used in this study.
