# Supplementary material for: O-Polysaccharides of LPS Modulate E. coli Uptake by Acanthamoeba castellanii
Source: Microorganisms. 2023 May 24;11(6):1377. doi: 10.3390/microorganisms11061377 (PMC10304059; doi:10.3390/microorganisms11061377)
Supplement: Supplementary file 1 [file microorganisms-11-01377-s001.zip › microorganisms-2372304-supplementary.pdf]

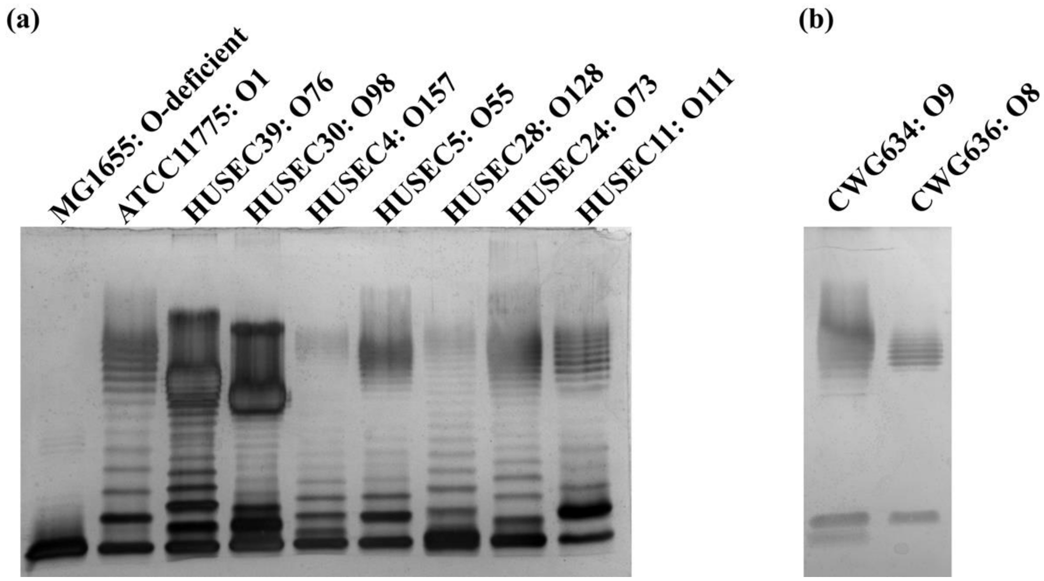

Figure S1. **(a)** LPS was extracted using hot phenol from *E. coli* strains MG1655, ATCC11775, HUSEC39, HUSEC30, HUSEC4, HUSEC5, HUSEC28, HUSEC24, HUSEC11. Purified LPS was visualized on 13% SDS-PAGE followed by silver staining. **(b)** LPS was extracted using hot phenol from *E. coli* strains CWG634 and CWG636 in LB supplemented with 0.04% Mannose. Purified LPS was visualized on 14% SDS-PAGE followed by silver staining.
